# Retraction: The data dimensionality reduction and bad data detection in the process of smart grid reconstruction through machine learning

**DOI:** 10.1371/journal.pone.0319594

**Published:** 2025-03-05

**Authors:** The PLOS One Editors

The *PLOS One* Editors retract this article [1] because it was identified as one of a series of submissions for which we have concerns about peer review integrity and potential manipulation of the publication process. These concerns call into question the validity and provenance of the reported results. We regret that the issues were not identified prior to the article’s publication.

All authors either did not respond directly or could not be reached.
